# Inequalities in Tobacco Retailer Compliance Violations Across the State of Oklahoma, 2015–2019

**DOI:** 10.1007/s10900-022-01091-7

**Published:** 2022-04-27

**Authors:** Ami E. Sedani, Sixia Chen, Jessica E. Beetch, Sydney A. Martinez, Hanh Dung N. Dao, Janis E. Campbell

**Affiliations:** Department of Biostatistics and Epidemiology, Hudson College of Public Health, University of Oklahoma Health Sciences Center, 801 NE 13th Street, 73104 Oklahoma City, OK United States

## Abstract

**Objective:**

To evaluate the relationship between compliance check violations, and characteristics of the tobacco retailer and neighborhood social vulnerability in Oklahoma.

**Design:**

This cross-sectional study utilized the US Food and Drug Administration (FDA) Compliance Check Inspections of Tobacco Product Retailers database for 2015–2019. These data were combined with Neighborhood social vulnerability variables using the Centers for Disease Control and Prevention (CDC) Social Vulnerability Index.

**Setting:**

The setting of this study is the state of Oklahoma, USA.

**Outcome measures:**

The outcome variable for this analysis was whether a sale was made to the youth during the compliance check (e.g., violation; yes/no) regardless of the outcome of the violation, and number of violations per a retailer.

**Results:**

We observed a strong association between having a violation and retailer store type, after controlling for socioeconomic vulnerability and percentage of mobile homes. The proportion of a tobacco retailer’s violations also varied by store type.

**Conclusions:**

More targeted enforcements and retailer education by store type may be necessary to increase compliance.

## Introduction

Despite significant progress in tobacco control and prevention efforts, tobacco use remains the leading cause of preventable disease and death in the U.S.[1] Preventing tobacco initiation among youth is a critical component of comprehensive tobacco control programs, with 90% of adult daily smokers reporting beginning smoking before they turned 19 years old.[2] Surveillance and evaluation of age-restriction policies, often done through compliance checks at tobacco retailers, are important to closely monitoring progress towards preventing initiation among young, and identify any inequalities in compliance.

A 2016 systematic review found that the likelihood of a sale to a minor (e.g., compliance check violation) varies by race, ethnicity and gender of the minor; however, results regarding the direction and magnitude of neighborhood characteristics predicting violations have been contradictory.[4] There has been further investigation regarding the store-level attributes in combination with the neighborhood characteristics.[5, 6] Variation in findings may be a result of differences in methodology as well as changes in tobacco use and regulation over time. Alternatively, these factors related to compliance check violations can be explained through the lens of a larger environmental and social context referred to as the Social Determinants of Health (SODH).[7] These determinants and their distribution in a given area impact health disparities, especially for the marginalized populations; therefore, models need to consider cumulative risk, not just one social risk factor.[8] Holistic index-based approaches, such as the social vulnerability index (SVI), have been proposed as a multi-tiered approach to understanding and addressing the SDOH factors.

Although existing literature examines individual-level predictors, we extend the literature by conducting the first study to examine the relationship between a composite measure of overall social vulnerability and compliance check violations. In an exploratory analysis, we sought to evaluate the relationship between compliance check violations and characteristics of the tobacco retailer (including neighborhood social vulnerability) by census tract in Oklahoma using the FDA Compliance Check Inspections of Tobacco Product Retailers (CCIT) database for 2015–2019. Given that tobacco control resources are limited, it is imperative that we design efficient compliance check programs focusing on targeting high-risk retailers.

## Methods

### Setting and Study Design

Reporting of this study followed the Strengthening the Reporting of Observational Studies in Epidemiology (STROBE) checklist.[9] A cross-sectional design was used to evaluate the relationship between compliance check violations and characteristics of the tobacco retailer. The setting of this study is the state of Oklahoma, which is a state in the South Central region of the US, and is 69,899 square miles (177,660 km^2^). It has a population of around 3.9 million (2019), and has the second largest population of American Indians/Alaskan Natives in the nation. For decades, tobacco industry lobby have directly interfered in Oklahoma lawmaking.[10] However, progress has been achieved despite very strong preemption clauses written into Oklahoma’s state tobacco laws.[11].

### Data Sources

To assess youth tobacco violations, we used the data from the publically available FDA CCIT database for October 2015 through September 2019 (e.g., FDA fiscal year 16–19) for Oklahoma. The data contain retailer name, street address, if a minor (less than 18 years old) was involved, if a sale was made, inspection date, inspection result, and charges (if any). States are required to develop a formal sampling strategy that inspects 20% or more of the tobacco retailers in the state each year; however, states may implement their sampling plans and inspections in different ways. The FDA does not release the compliance inspection sampling methodology including weights.

Neighborhood social vulnerability predictor variables for each tobacco retailer were obtained using the Centers for Disease Control and Prevention (CDC) SVI values for 2018 from the CDC’s Agency for Toxic Substances and Disease Registry’s Geospatial Research, Analysis, and Services Program.[12] The SVI indicates the relative vulnerability of every U.S. census tract, utilizing the American Community Survey (ACS) for 2014–2018 (5-year). The variables included in SVI were selected based on an extensive review of the literature and represent socioeconomic and demographic characteristics within four themes: [1] socioeconomic status, [2] household composition, [3] minority status and language, and [4] housing and transportation. The overall index is scored from 0 to 1, with higher values denoting higher vulnerability. We then categorized geographic location type for tobacco retailers into four categories (urban – metropolitan; suburban; large town; small town) using the rural-urban commuting area (RUCA) codes from the United States Department of Agriculture (USDA) which uses measures of population density, urbanization, and daily commuting patterns.[13].

### Data Preparation

Data for each fiscal year were merged into a single database. To handle locations that may have had multiple observations, we linked records by name and street address yielding a final sample size of 3,739 unique tobacco retailers. New variables were created including a deduplication ID, total number of inspections during the time-period, number of failed inspections, and if an inspection was ever failed.

The unique tobacco retailers were then classified by store type to assess if violations differed by store type. If the store type was not obvious from the outlet name, this was determined by searching internet directories for further information on the outlet. If a location was unable to be found by one researcher, two more attempted to find the location, resulting in all geocoded locations being classified. A hierarchy methodology was used to classify locations that were mixed-use. We consolidated store types into seven initial categories: [1] gas station and/or convenience store, [2] supermarket/grocery store, [3] warehouse club/supercenter, [4] tobacco store, [5] liquor store, [6] pharmacy/drug store, and [7] other. The “other” category included bar or club; recreational location (e.g. golf course, casino, pool hall, etc.); eating establishment; hotel and campground; and miscellaneous store (e.g. trucking, tire store, fishing store, pawnshop, etc.). The quality of the work was assessed and verified collectively by two of the authors by randomly checking 1% of the processed results.

Tobacco retailer locations were geocoded using ArcGIS Version 10.8.1 (ESRI, Redlands, CA) Batch Geocode methods. All of the available addresses were successfully geocoded, indicating a high success rate. Each geocoded retailer was then joined with census block groups to then join with the SVI data using Federal Information Processing Standard (FIPS) codes.

### Statistical Analysis

All statistical analyses were conducted using SAS v. 9.4 (SAS Institute, Cary, NC). Descriptive statistics were first used to determine compliance check violations. The outcome variable for this analysis was whether a sale was made to the youth during the compliance check (e.g., violation) regardless of the outcome of the violation. We examined the distribution of the covariates of interest for the entire sample and for retailers with at least one violation during the study period. Due to low numbers of compliance checks in certain store type categories, the store type variable was reduced to five groups. Logistic regression was used to obtain the Prevalence Odds Ratios (PORs) for having at least one violation. Variables that were statistically significant, with p-value of ≤ 0.10, in the bivariate analysis were included in the final model building. If two covariates were highly correlated, we left one out of the model given that they are likely to account for the same variability in the outcome.

We used multivariable logistic regression models to study the association of retailer characteristics and neighborhood social vulnerability, and the dichotomous outcome using manual stepwise variable selection, a semi-automated process. Potential statistical interaction was investigated by adding two-way interaction terms for each variable considered in the final model. Interaction terms that were significantly associated with the outcome were retained in the final model. The analyses were repeated with stratification by store type due to the presence of effect modification. Adjusted PORs and corresponding 95% confidence intervals (CIs) were calculated, and a two-sided p-value of ≤ 0.05 was considered statistically significant. Differences in the results were also evaluated by examining changes in PORs. To aid in interpretability, we then conducted pairwise comparisons among the predictor variable retailer store type to describe the pattern of mean differences.

We then created a new continuous outcome, the proportion of compliance checks that a retailer failed, and subset the data to only include retailers that had a proportion of failed compliance checks greater than 0 and less than 1. Multivariable linear regression models using PROC GLM procedure in SAS were conducted to predict the logit-transformed proportion of violations, and retailer and neighborhood social vulnerability characteristics. The logit transformation respects the range of the proportion and makes the transformed distribution closer to normal distribution. Variables selection and interaction terms were determined using methods mentioned above. To confirm normality and the appropriate use of the linear model, a Q–Q plot and Kolmogorov–Smirnov test were conducted.

## Results

### Descriptive Statistics

Out of the 6,604 compliance checks completed from 2015 to 2019, there were 3,379 (51.17%) unique locations. Characteristics of unique locations and compliance check failure are presented in Table 1. The majority of locations were gas stations/convenience stores (53.54%), and the majority had multiple locations or were affiliated with a chain (55.18%). There were a total of 1,084 (28.99%) stores that were ever issued a violation of any kind. Although more than half of the retailers in the sample were convenience stores, they only accounted for 32.02% of locations ever having a violation. Moreover, tobacco stores were only 6.98% of the sample, but accounted for 38.31% of locations ever having a violation. The number of checks per vendor ranged from one to eight during the study period with only 18.75% (n = 701) of locations having three or more compliance checks. As the number of compliance checks at a retailer location increased, so did their probability of having a violation, indicating that if a location fails a compliance check they are more likely to have more compliance checks (data not shown). The crude odds of violation were 1.50 (95% CI: 1.30, 1.73) times higher among independently owned retailers compared to chain retailers (p < 0.0001). Violations differed significantly by store type (p < 0.0001), ranging from 17.20% (supercenters and pharmacies) to 38.31% (tobacco stores).

**Table 1:** Descriptive statistics for compliance check violations (e.g., ever sold to minor) by Retailer and neighborhood social vulnerability, Oklahoma, 2015–2019

	Total Sample (n = 3739)			One or More Violations (n = 1084)			
	**N**	**%**		**N**	**%**	**POR (95% CI)**	**P-value**
**Retailer Characteristics**							< 0.0001*
**Store Type**							
Convenience Stores	2002	53.54		641	32.02	2.27 (1.83, 2.81)	
Grocery Stores	452	12.09		123	27.21	1.80 (1.36, 2.39)	
Supercenters, and Pharmacies	721	19.28		124	17.20	Ref.	
Tobacco Stores	261	6.98		100	38.31	2.99 (2.18, 4.10)	
Other	303	8.10		96	31.68	2.23 (1.64, 3.04)	
**Retailer Affiliation**							< 0.0001*
Chain	2063	55.18		521	25.25	Ref.	
Independent	1676	44.82		563	33.59	1.50 (1.30, 1.73)	
**No. of Compliance Checks**							< 0.0001*
1	2030	54.29		196	9.66	Ref.	
2	1008	26.96		266	26.39	3.35 (2.74, 4.11)	
3+	701	18.75		622	88.73	73.67 (55.87, 97.15)	
**Neighborhood Social Vulnerability**							
**Urban-Rural Classification** ^a^							0.1289
Urban	1518	40.60		420	27.67	0.92 (0.72, 1.17)	
Suburban	404	10.81		119	29.46	Ref	
Large Town	930	24.87		261	28.06	0.93 (0.72, 1.21)	
Small Town	887	23.72		284	32.02	1.13 (0.87, 1.47)	
	**Mean**	**STD**		**Mean**	**STD**	**OR (95% CI)**	**P-value**
**Overall SVI Rank**	56.69	27.30		56.52	27.07	1.17 (0.90, 1.52)	0.2320
**Socioeconomic Vulnerability**	53.82	27.20		55.52	26.90	1.39 (1.07, 1.80)	0.0140*
Below Poverty	18.20	10.21		18.69	10.51	1.01 (1.00, 1.01)	0.0629*
Unemployed	5.84	3.49		5.94	3.40	1.01 (0.99, 1.03)	0.2947
Income, $	25256.46	9185.74		24910.67	9430.54	1.00 (1.00, 1.00)	0.1373
HS Diploma	14.16	8.42		14.76	8.64	1.01 (1.00, 1.02)	0.0062*
**Household Composition Vulnerability**	54.93	28.16		55.07	27.52	1.02 (0.80, 1.32)	0.8523
65 and older	15.43	5.21		15.38	5.25	1.00 (0.98, 1.01)	0.7168
Aged 17 or Younger	24.34	5.30		24.46	5.41	1.01 (0.99, 1.02)	0.3923
Disability	17.40	5.54		17.45	5.30	1.00 (0.99, 1.02)	0.7085
Single Parent	10.27	4.93		10.17	4.93	0.99 (0.98, 1.01)	0.4307
**Minority Status Vulnerability**	50.67	28.88		50.55	29.80	0.98 (0.77, 1.25)	0.8723
Minority	35.05	17.06		35.53	17.65	1.00 (1.00, 1.01)	0.2675
Speaks English “Less than Well”	2.37	4.03		2.55	4.25	1.02 (1.00, 1.03)	0.0804*
**Housing Type & Transportation Vulnerability**	57.27	27.36		57.54	26.80	1.05 (0.81, 1.36)	0.7015
Multiunit	6.46	10.67		6.09	10.03	1.00 (0.99, 1.00)	0.1758
Mobile Homes	9.45	10.27		10.04	10.53	1.01 (1.00, 1.02)	0.0265*
Crowding	3.08	2.58		3.24	2.72	1.03 (1.01, 1.06)	0.0163*
No Vehicle	6.28	5.08		6.34	5.18	1.00 (0.99, 1.02)	0.6106
Group Quarters	2.69	6.78		2.63	6.48	1.00 (0.99, 1.01)	0.7053
**Other**: Uninsured	15.83	6.93		16.27	7.01	1.01 (1.00, 1.02)	0.0154*

**Notes**: Vulnerability index is scored from 0 to 1 with higher values denoting higher vulnerability.

^a^Urban Core: contiguous built-up areas of 50,000 people or more. Suburban: areas, often in metropolitan counties, with primary high commuting flows to urban cores and all other areas with secondary commuting flows of 30-49% of the population to urban cores. Large Town: towns with populations of 10,000–49,999 and surrounding rural areas with 10% or more primary commuting flows to these towns, and towns with secondary commuting flows of 10% or more to Urban Cores. Small Town/Rural Areas: towns with populations below 10,000 and surrounding commuter areas with more than a one-hour driving distance to the closest city.

*statistically significant at ≤ 0.10.

Regarding neighborhood social vulnerability, although the highest amount of compliance checks occurred in urban census tracts (40.60%), small towns had the highest percentage of violations (32.02%). Similarly, only 10.81% of the locations in our same were located in suburban areas, but they accounted for 29.46% of of locations ever having a violation. When examining SVI, there was only a small deviation from the national average of 50 for overall SVI rank and all social vulnerability themes. The overall SVI rank was not significantly associated with a retailer having a violation (p = 0.232). The association between social vulnerability themes and retailer violation were further explored. Among the four themes that compromise the overall SVI rank, only one social vulnerability theme (socioeconomic vulnerability) was found to be associated with having a violation before adjusting for any other covariates (p = 0.014). Retailers that had a violation were in census tracts with higher socioeconomic vulnerability (POR = 1.39, 95% CI: 1.07, 1.80). Specifically, among the socioeconomic status indicators, the percent of individuals below poverty (p = 0.0629), and the percentage of individuals with less than a high school diploma were found to be a significant predictor (p = 0.0062); however, these two variables were found to be highly correlated (r^2^ = 0.80). Among minority status indicators, the only variable associated with violations was the percentage of individuals who speak English “Less than Well” (p = 0.0804). Among housing type and transportation status indicators, violations were likely to occur in stores located in census tracts with a greater percentage of mobile homes (p = 0.0265), and a greater percentage of more people than rooms (e.g., crowding) (p = 0.0163). The percentage of individuals without health insurance was also found to be associated with retailer violation (p = 0.0154).

### Multivariable Logistic Regression

Retailer store type, retailer affiliation, socioeconomic vulnerability, the percentage of individuals with at least a high school diploma, the percentage of households that speak English “less than well”, the percentage of mobile homes, the percentage of households with crowding, and the percentage of individuals without health insurance were all entered into the multivariable model. Formal testing revealed statistical interaction between the percentage of mobile homes and retailer store type (p_interaction_ = 0.0196). However, socioeconomic vulnerability was not associated with retailer violation (p_interaction_ = 0.6970). The final multivariable logistic regression model is presented in Table 2, for 3,379 unique retailer locations. Ever having a violation was significantly associated with retailer store type, after controlling for socioeconomic vulnerability, percentage of mobile homes, and the interaction term of percentage of mobile homes and store type (p = 0.0003).

**Table 2:** Adjusted prevalence odds ratios (aPOR) for association between having a compliance check violation and retailer characteristics (n = 3,739).*

	Adjusted POR (95% CI)	p-value
**Store Type**		0.0003^†^
Convenience Stores	1.05 (0.90, 1.23)	
Grocery Stores	0.85 (0.66, 1.09)	
Supercenters, and Pharmacies	Ref.	
Tobacco Stores	1.59 (1.22, 2.06)	
Other	1.06 (0.79, 1.42)	
**Socioeconomic Vulnerability**	1.46 (1.12, 1.90)	0.0056^†^
**Per. Mobile Homes**	1.00 (0.99, 1.01)	0.5633
Per. Mobile Homes *Store Type		0.0196^†^
Convenience Stores	1.01 (1.00, 1.02)	
Grocery Stores	1.01 (0.99, 1.03)	
Supercenters, and Pharmacies	Ref.	
Tobacco Stores	1.00 (0.98, 1.03)	
Other	1.01 (0.99, 1.03)	

**Notes**:

*Adjusted for: store type, socioeconomic vulnerability, percentage of mobile homes, and percentage of mobile homes*store type

^†^statistically significant at ≤ 0.05

To aid in interpretability, we also conducted pairwise comparisons among the predictor variable conditioning on the average percent mobile homes, which is 9.45% (data not shown). The following comparisons were not significant: convenience stores vs. “other” (p = 0.8563), tobacco stores vs. “other” (p = 0.0717), and “other” vs. grocery stores (p = 0.1790). The following comparisons were significant: convenience stores vs. tobacco stores (p = 0.0336), convenience stores vs. grocery stores (p = 0.0343), convenience stores vs. supercenters and pharmacies (p < 0.0001), tobacco stores vs. grocery stores (p = 0.0014), tobacco stores vs. supercenters and pharmacies (p < 0.0001), “other” stores vs. supercenters and pharmacies (p < 0.0001), and grocery stores vs. supercenters and pharmacies (p < 0.0001). Tobacco stores were more likely to have a compliance check violation than supercenters and pharmacies (aPOR = 3.19, 95% CI: 2.27, 4.49), and more likely than grocery stores (aPOR = 1.76, 95% CI: 1.24, 2.49), after adjusting for the average percent mobile homes. Similarly, convenience stores were more likely to have a violation than supercenters and pharmacies (aPOR = 2.32, 95% CI: 1.86, 2.89), and more likely than grocery stores (aPOR = 1.28, 95% CI: 1.02, 1.61). On the other hand, grocery stores were more likely to have a violation than supercenters and pharmacies (aPOR = 1.82, 95% CI: 1.36, 2.42). The odds of violation were 2.26 (95% CI: 1.65, 3.11) times higher for “other” stores compared to supercenters and pharmacies, after adjusting for the average percent mobile homes.

### Multivariable Linear Regression

There were a total of 840 retailers included in the liner regression analysis with a mean proportion of failed compliance checks of 0.42 (STD = 0.12). The following variables were not significant: retailer affiliation (p = 0.0806), socioeconomic vulnerability (p = 0.3248), the percentage of individuals with at least a high school diploma (p = 0.9272), the percentage of households that speak English “less than well” (p = 0.9957), the percentage of mobile homes (p = 0.2115), the percentage of households with crowding (p = p = 0.1859), and the percentage of individuals without health insurance (p = 0.6905). Retailer store type was the only significant predictor (p = 0.0006). The results of the final linear regression model are presented in Table 3. The average of the logit of the proportion of violations increases by 0.22 for convenience stores compared to superstores and pharmacies. The average of the logit of the proportion of violations increases by 0.17 for tobacco stores compared to superstores and pharmacies, and by 0.17 for “other” stores compared to superstores and pharmacies.

**Table 3:** Estimates and standard errors from linear regression model for the probability of a violation (n = 840).*

	Estimate	SE	p-value
**Store Type**			0.0006^†^
Convenience Stores	0.22	0.06	
Grocery Stores	0.06	0.07	
Superstores & Pharmacies	Ref.		
Tobacco Stores	0.17	0.08	
Other	0.17	0.08	

**Notes**: Model fit: r^2^ = 0.0234

*Adjusted for store type

^†^statistically significant at ≤ 0.05

## Discussion

This paper explored the relationship between compliance check violations and characteristics of the tobacco retailer (including neighborhood social vulnerability) utilizing a large sample of retailers within a state that has a historically high prevalence of tobacco-use. Investigation of this relationship is imperative for informing policy and subsequently ensuring successful implementation of minimum legal sales age (MLSA) policies. [14–17, 18, 19]We observed a strong association between having a violation and retailer store type, as well as proportion of violations and store type. This finding is consistent with previous studies.[5, 6, 20] When examining self-reported sources of youth access to tobacco products, those who attempted to buy tobacco or gave money to someone else most commonly purchased tobacco products from convenience stores and/or gas stations.[21] Similarly, these findings are reflective of a 2021 study in Oklahoma investigating storefront smokeless tobacco advertising, which found that store type was highly associated with advertising.[22] Although, evidence regarding the importance of retailer store type appears to be strengthening over time, it is important to note that store type is not included in the FDA CCTI database, and future work may greatly benefit from the addition of this variable.

Contrary to previous literature, racial and ethnic minority status was not associated with a compliance check violation in the context of this study.[14–17] Instead, our study observed a stronger relationship between retailers located in areas with a greater share of socioeconomic disadvantage and disproportionate differences in compliance check violations. On that same note, perhaps the most complex issue is the interrelationship between retailer store type and the demographic characteristics of a given neighborhood, such as percentage of mobile homes and socioeconomic vulnerability. The explanation for the potential interaction between store type and percentage of mobile homes is not readily apparent and may be spurious, although there are a few possible explanations. One reason may be that the presence of certain retailer store types vary depending on neighborhood demographics, which has been observed in neighborhood food availability research, and more recently tobacco retailer density research.[23, 24] Specifically, mobile homes have been found to be more concentrated in less densely populated areas with higher prevalence of poverty, and further away from health services.[25] Outdated zoning codes may promote inequalities in the built and social environments. Further research is needed to disentangle the individual and joint effects of these factors in order to better understand the drivers of social stratification and marginalization to subsequently advance equitable change. Regardless of the cause of these inequities, disparities in neighborhood composition has important social justice implications.

There are several key limitations that should be considered when interpreting the results of the current study. First, due to the cross-sectional nature of this study, we had insufficient information regarding the temporal sequence; thus, conclusions cannot be drawn regarding causation. However, purposes of this study were not to establish causality, and instead were to inform planning and resource allocation, and for hypothesis generation. It is also important to note that cross-sectional studies represent a single point in time, and our study was conducted before Dec. 20, 2019, when the federal minimum age for sale of tobacco products was raised from 18 to 21 years. This legislation (known as “Tobacco 21” or “T21”) became effective immediately. Thus, these data reflect compliance among under 18 at the time of an 18 minimum age. Second, there are numerous concerns inherently related to the utilization of compliance checks databases; thus, incomplete control for confounders might have influenced our results. There are a number of factors that cannot be accounted for with available data, including characteristics of the buyer, length of retailer existence, circumstances surrounding the transaction, and the sampling strategies used.[26] Similarly, there is also the concern that retailers may only sell to minors that they know, and currently the FDA CCIT database does not allow us to consider this relationship. Lastly, we did not account for spatial correlations; however, there currently is not a gold standard or agreement on how to account for both within- and between- tract correlations. Despite limitations, our study has important findings and methods that add to current policy discussions regarding preventing tobacco initiation among youth and young adults. Of note, a novel aspect of the current study was the use of the CDC’s SVI database. Although traditionally used for emergency preparedness planning, applications of the SVI have also been used explore the relationships between social vulnerability, and health behaviors and outcomes.[27–30] These results also add to the growing body of evidence that implementing tools to assess SVI or other multifaceted components of SDOH, may provide valuable insight regarding how to better allocate scarce public health resources and narrow health inequities.

## Conclusions

Building on previous work, our study found a relatively high rate of sales to underage buyers, and subsequently, differences by retailer store type considering socioeconomic status and percentage of mobile homes. These results demonstrate the need for a more targeted approach to compliance checks, and continued initiatives aimed at preventing tobacco initiation among youth and young adults. Even in a state that has adopted strong youth access laws, opportunities for improvement still exist. This is particularly important with the presence of the electronic nicotine delivery systems (ENDS) epidemic among teens, and the current pandemic-related changes in tobacco use where we have seen increases in cigarette use among youth.[31, 32].

## Implications for Policy & Practice

- The findings demonstrate compliance check violations vary by retailer store type, socioeconomic vulnerability, and percentage of mobile homes in Oklahoma. Thus, more targeted enforcements and retailer education by store type may be necessary to increase compliance.
- It is important to continue to monitor tobacco retailer compliance check violations to identify potential health inequities, especially now that the age for purchasing tobacco has been raised to 21 years.

## Data Availability

The raw data are openly available in the FDA Compliance Check Inspections of Tobacco Product Retailers database at Compliance Check Inspections of Tobacco Product Retailers (through 08/31/2021) (fda.gov). Derived data supporting the findings of this study are available from the corresponding author [A.E.S] on request.

## Abbreviations

POR: Prevalence Odds Ratio
CI: Confidence Interval
No.: Number
HS: High School
STD: Standard Deviation
SVI: Social Vulnerability Index
